# Impact of pre-pandemic sick leave diagnoses on the length of COVID-19-related sick leave: a nationwide registry-based study

**DOI:** 10.1186/s12889-023-15115-x

**Published:** 2023-01-29

**Authors:** Tamar Abzhandadze, Emma Westerlind, Hanna C. Persson

**Affiliations:** 1 Institute of Neuroscience and Physiology, the Sahlgrenska Academy, University of Gothenburg, Gothenburg, Sweden; 2 Department of Occupational Therapy and Physiotherapy, Sahlgrenska University Hospital, Gothenburg, Sweden; 3 Division of Clinical Geriatrics, Department of Neurobiology, Care Sciences and Society, Karolinska Institutet, Stockholm, Sweden

**Keywords:** Epidemiology, Return to work, Sick leave, Registry-based study, SARS-CoV-2 infection

## Abstract

**Background:**

The COVID-19 pandemic has caused difficulties and changes in many aspects of people’s health and lives. Although infection affected work capacity, during the first wave policies for sick leave due to COVID-19 were unclear. The aim of this study was to investigate the impact of sick leave diagnoses in the year before the COVID-19 diagnosis  on sick leave duration due to COVID-19 in a nationwide non-hospitalised population.

**Methods:**

Data from three Swedish registries were analysed for sick leave commencing between 1 March and 31 August 2020, with a follow-up period of 4 months. Sick leave due to COVID-19 was considered the number of days that sickness benefits were used and included at least one registered COVID-19 diagnosis. Sick leave in the year before COVID-19 diagnosis were categorised into five diagnostic groups and one reference group (participants without prior sick leave).

**Results:**

The study comprised 8935 individuals who received sickness benefits due to COVID-19 in Sweden during the first pandemic wave (mean age 46.7 years, 67% females, and 24% had diagnoses for sick leave in the year before COVID-19 diagnosis). The duration of sick leave due to COVID-19 was significantly higher in the groups with prior sick leave owing to musculoskeletal system diseases (odds ratio [OR]: 1.08, 95% confidence interval [CI]: 1.01–1.15); respiratory system diseases (OR: 1.22, 95% CI: 1.14–1.31); all other isolated diagnoses (OR: 1.08, 95% CI: 1.03–1.14); and multiple diagnoses (OR: 1.32, 95% CI: 1.21–1.43).

**Conclusions:**

The results of this nationwide registry-based study indicate that individuals with premorbid conditions are more prone to longer sick leave durations due to COVID-19. Prediction of sick leave duration during the first wave of the COVID-19 pandemic is complex and several factors played a role.

**Supplementary Information:**

The online version contains supplementary material available at 10.1186/s12889-023-15115-x.

## Background

The Public Health Agency of Sweden announced the outbreak the coronavirus disease 2019 (COVID-19) on 10 March 2020 [1]. During the first wave of the COVID-19 pandemic (March to September 2020), many people refrained from using healthcare services owing to the high risk of infection or to avoid overloading the healthcare system. Moreover, population testing for the severe acute respiratory syndrome coronavirus 2 (SARS-CoV-2) was not implemented at the beginning of the first wave [1]. Thus, the diagnosis of SARS-CoV-2 infection could be based on positive laboratory test results or clinical symptoms according to the World Health Organization criteria and International Statistical Classification of Diseases (ICD-10) emergency codes for COVID-19.

The COVID-19 pandemic has caused difficulties and changes in many aspects of people’s health and lives [2, 3], and affected work capacity [4]. However, policies for sick leave due to COVID-19 were unclear, with large variations among countries that offer paid sick leave [5, 6]. In Sweden, compensation for sick leave is tax-financed and is thus regulated by the Swedish Social Insurance Agency (SSIA). According to the SSIA, the number of sick leave cases initiated between March and May 2020 were double that of the previous year [7].

We previously reported that among individuals who started sick leave for COVID-19 during the first wave of pandemic in Sweden, 9% of individuals with COVID -19 used at least 4 months of sick leave [8]. Individuals requiring inpatient care and individuals with a previous history of sick leave had high odds of taking ≥ 84 days of sick leave due to COVID-19 [8]. Moreover, a higher proportion of hospitalised individuals had the persistent symptoms of long COVID-19 [9] that could theoretically lead to increased duration of sick leave. In addition, several studies have found that female sex, older age, and certain health conditions are related to a longer duration of sick leave [10, 11]. A policy brief that included evidence-based studies on long COVID-19 (i.e., reporting symptoms of COVID-19 for at least 12 weeks) showed that hospitalized patients had a higher prevalence of long COVID-19 [9]. The association between previous sick leave diagnoses and sick leave duration due to COVID-19 in individuals who do not require inpatient care remains unclear [9]. Moreover, one large study found differences in self-reported symptoms after COVID-19 diagnosis in individuals with and without a positive SARS-CoV-2 test result and self-reported symptoms such as joint pain, breathing difficulties, digestive tract problems, and cognitive impairment [12]. However, possible differences between individuals with a laboratory-confirmed SARS-CoV-2 infection and individuals with a clinical symptom-based diagnosis regarding sick leave due to COVID-19 remains unclear.

The primary aim of this study was to determine the impact of sick leave diagnoses in the year before the COVID-19 diagnosis on the sick leave duration due to COVID-19. Our secondary aim was to identify the possible factors that affect sick leave duration in individuals with a COVID-19 diagnosis, with and without SARS-CoV-2 detection. These aims were addressed by analysing national Swedish registry data on individuals with COVID-19 who did not require inpatient care during the first wave of the COVID-19 pandemic.

## Methods

### Study design and procedure

In this registry-based study, we analysed data from three national registries: the SSIA, which comprises data on employment status and type, sick leave, and sickness benefits; the Swedish National Board of Health and Welfare registry, which contains data on causes of death during the study period and inpatient care; and Statistics Sweden, which contains sociodemographic data of all people registered in Sweden. A unique Swedish personal identification number was used to pool the data. The data files were pseudonymised and contained serial numbers. The code key for the serial numbers was maintained by the registry holders.

### Study population

Residents of Sweden aged ≥ 18 years who received sickness benefits due to COVID-19 (defined according to the ICD codes, including virus identified [U07.1] or virus not identified [U07.2]) were included in this study. Sick leave was required to have commenced between 1 March and 31 August 2020, with a follow-up period of 4 months. Individuals who required inpatient care or died during the study period were excluded.

### Definitions of major concepts

The COVID-19 diagnosis included individuals with and without confirmation of SARS-CoV-2 infection because mass testing had not been implemented in Sweden during the first wave of the pandemic. If SARS-CoV-2 was identified and the virus was confirmed by laboratory tests, the ICD code U07.1 was assigned regardless of the severity of the clinical signs or symptoms. If SARS-CoV-2 was not identified (ICD code U07.2), COVID-19 was diagnosed clinically or epidemiologically in the absence of laboratory tests.

Sick leave was defined as receiving sickness benefits, regardless of the amount. Sickness benefits can be granted by the SSIA to anyone who has been working in Sweden (both employed and self-employed), and during parental leave, active studies, and registered unemployment [8]. The employer provides sick pay for the first 2 weeks of absence from work due to sickness. From the 15th day onwards, the SSIA pays the sickness benefits. For the purpose of this study, if individuals received sick pay for 2 weeks followed by sickness benefits from the SSIA, the sick pay period was included in the total sick leave period. For unemployed individuals, sickness benefits are provided from the start of the sick leave [8].

### Variables

The sick leave period due to COVID-19 was counted as the number of days with sickness benefits, and each individual had to have at least one registered COVID-19 diagnosis [8]. Other predefined related diagnoses were merged with COVID-19 sick leave if the gap in non-registration between the sick leave periods was ≤ 2 weeks. The related diagnoses were unspecified virus infections, fever, or a second sick leave registration because of a COVID-19 diagnosis [8]. The gap of 14 days was chosen for several reasons. For example, the physician might not have had time to provide the patient with a sick leave certificate, or the person might have tried to go to work but found that they were unable to work and needed more time for recovery.

If sick pay was received in the first 14 days, it was included in the sick leave period. Two sick leave periods were merged if they were separated by a gap of ≤ 14 days. The sick leave lasted for a minimum of 1 day and a maximum of 122 days (corresponding to a 4-month follow-up period after the initial diagnosis). Moreover, individuals were considered to have long COVID if the length of sick leave was ≥ 84 days [9].

Sick leave before COVID-19 was defined as being on sick leave for at least one 28-day period between 1 March 2019 and the date of the first COVID-19 sick leave registration [8]. Five groups were created to make the results interpretable: group 1 included diagnostic codes corresponding to mental, behavioural, and neurodevelopmental disorders (ICD - F codes); group 2 included diagnostic codes corresponding to musculoskeletal system and connective tissue diseases (ICD - M codes); group 3 included diagnostic codes corresponding to respiratory system diseases (ICD - J codes); group 4 included all other isolated diagnostic codes (except for ICD codes - F, M, and J); and group 5 included individuals who were on sick leave during the year before the COVID-19 diagnosis for multiple diagnosis codes. We included one reference group ‘0’ which included individuals without sick leave during the year because their COVID-19 diagnosis. All sick leave diagnoses were included in the analysis regardless of the sick leave duration.

Employment status was defined as employed (including temporary parental leave and combined self-employment and employment), self-employed, and unemployed (including students) [8]. The income variable was based on the disposable income per person in 2019 and was counted in thousands of Swedish Krona (average exchange rates during 2020 according to the Swedish Riksbank: 1 EUR = 10.5 SEK, 1 GBP = 11.8 SEK). Education level was divided into four strata. The country of birth was categorised into five groups. Marital status was classified as married, single, divorced, and widow/widower [13]. Variables on children living at home and children aged ≤ 18 years were included in the analysis. Detailed information on the variables used in the study, their codes, and their roles are provided in Supplementary Table S1.

### Statistical analysis

The data are presented as means and standard deviations (± SDs), medians and interquartile ranges (IQRs), and numbers with percentages (n, %). Non-parametric statistical tests were performed because many continuous variables had a skewed distribution. The chi-square test, Mann–Whitney *U* test, and Kruskal–Wallis test were used to compare the included and excluded individuals, and for intergroup comparisons based on COVID-19 diagnostic modalities. Spearman’s rank order correlation test (r_s_) was used to explore the correlation between possible explanatory variables (Supplementary Table S1). The strength of the correlation was interpreted as weak (< ± 0.39), moderate (± 0.40 to ± 0.69), or strong (≥ ± 0.70) [14].

Regression analyses were performed to determine how a previous diagnosis could predict the duration of sick leave during the first 4 months of the COVID-19 diagnosis.

#### Selection of the study variables

The primary independent variable in this study was a diagnosis necessitating sick leave in the year before the COVID-19 diagnosis. The study population was stratified into five groups according to prior diagnosis, and an additional group without any prior sick leave was used as the reference group. The outcome variable was the duration of sick leave during the 4 months following the COVID-19 diagnosis. Based on variable availability in the dataset and clinical reasoning, eight variables were identified, of which six were selected using a directed acyclic graph (DAG), which facilitates the presentation of a parsimonious model (Supplementary Figure S1). Age, sex, marital status, education level, employment status, and sick leave length ≥ 28 days in the year before COVID-19 diagnosis were identified as important variables according to the DAG.

#### Choosing the regression model

The outcome was a numerical variable (range, 1–122 days); the mean and variance values were compared to select the appropriate regression model. The mean was lower than the variance (43.1 vs. 657.7), indicating a positive (overdispersion) distribution of the outcome variable. Furthermore, the outcome variable did not contain ‘0’ counts. Therefore, a negative binomial regression analysis was used with a link function log [15].

#### Fitting and evaluation of the regression models

Negative binomial regression analyses were performed to identify the predictors of the length of sick leave related to COVID-19. All explanatory variables identified by DAG were entered into the model. The results were evaluated as follows. At the variable level, we reported regression coefficients and standard errors for the coefficients (β [SE]), *p*-values, and 95% confidence intervals (CIs). The models were evaluated using the Akaike information criterion (AIC), omnibus test (*p* < 0.05 indicates good fit), and log-likelihood ratio test. The analyses were performed on all study participants and subgroups stratified according to COVID-19 diagnoses (with and without SARS-CoV-2 detection).

Data were processed using SPSS software (Version 28.0., IBM Corp., Armonk, NY, USA). The significance level for all statistical tests was set at an alpha of 5%.

## Results

### Study population

**Fig. 1:**
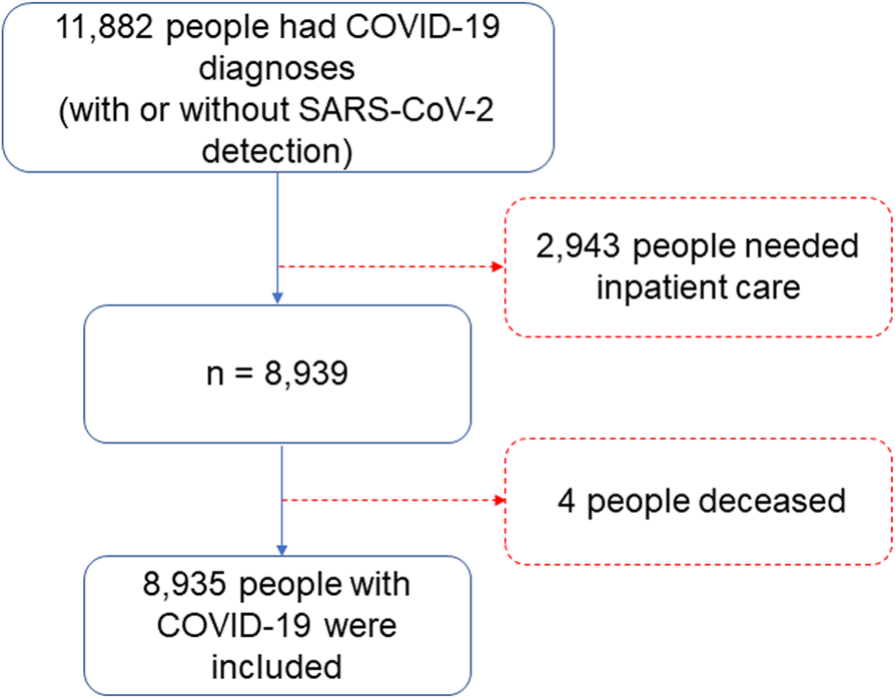
Flowchart of the study population

A total of 8935 individuals fulfilled the inclusion criteria (Fig. 1). The excluded (*n* = 2947) and included individuals (*n* = 8935) differed significantly by sex (*p* < 0.001) and age (*p* < 0.001). In the excluded group, 1886 (64%) were males and 1061 (36%) were females, mean age (SD) was 51.9 (9.9) years.

Detailed information on the study population is shown in Table 1. The mean age was 46.7 years, and the majority of the participants were female (67%). During the year before the COVID-19 diagnosis, 24% of the population had diagnoses related to sick leave, and 17% had taken ≥ 28 days of sick leave. Correlation analyses revealed a weak correlation between the majority of the variables (Supplementary Table S2).

**Table 1:** Characteristics of the study population (*n* = 8935)

Characteristics	Total (*n* = 8935)	COVID-19 virus not identified (*n* = 3833)	COVID-19 virus identified, (*n* = 5102)	*p*-value
Sociodemographic characteristics				
Sex, n (%)				** < 0.001**
Male	2911 (33)	1503 (39)	1408 (28)	
Female	6024 (67)	2330 (61)	3694 (72)	
Age, mean (± SD)	46.7 (11.4)	46.9 (11.3)	46.5 (11.5)	0.239^#^
Median (IQR)/min–max	48 (18)/18–76	48 (17)/19–76	48 (18)/18–75	
Country of birth, n (%)				** < 0.001**
Sweden	5943 (67)	2664 (70)	3279 (64)	
Nordic countries, except for Sweden	183 (2)	70 (2)	113 (2)	
European countries, except Nordic countries	833 (9)	309 (8)	524 (10)	
Asian countries	1317 (15)	523 (14)	794 (16)	
Other countries	653 (7)	264 (7)	389 (8)	
Education, n (%)				** < 0.001**
Primary school (≤ 9 years)	822 (9)	397 (10)	425 (8)	
Secondary school (10–12 years)	4456 (50)	1761 (57)	2695 (53)	
Short university education (13–14 years)	1269 (14)	595 (16)	674 (13)	
Long university education (≥ 15 years)	2324 (26)	1044 (28)	1280 (25)	
Income in 10,000 SEK, mean (± SD)	30.1 (24.8)	31.0 (35.0)	29.4 (12.5)	**0.001**^**#**^
Marital status, n (%)				**0.004**
Married	4087 (46)	1681 (44)	2406 (47)	
Single	3171 (36)	1385 (36)	1786 (35)	
Divorced	1552 (17)	716 (19)	836 (16)	
Widow/Widower	118 (1)	47 (1)	71 (1)	
Children living at home, yes, n (%)	4932 (55)	2092 (55)	2840 (56)	0.218
Age ≤ 18 years	3362 (38)	1436 (38)	1926 (38)	0.795
Sick leave prior to COVID-19 ≥ 28 days, yes, n (%)	1553 (17)	664 (17)	889 (17)	0.900
Diagnostic groups 1 year prior to COVID-19, n (%)				0.476
No prior sick leave	6568(76)	2834(76)	3734 (75)	
Mental, behavioural, and neurodevelopmental disorders	502 (6)	220 (6)	282 (6)	
Musculoskeletal system and connective tissue diseases	441(5)	180 (5)	261 (5)	
Respiratory system diseases	250 (3)	103 (3)	147 (3)	
All other isolated diagnoses	683 (8)	277 (7)	406 (8)	
Multiple diagnoses	232 (3)	109 (3)	123 (3)	
Employment status, n (%)				** < 0.001**
Employed	8645 (97)	3651 (95)	4994 (98)	
Self-employed	182 (2)	112 (3)	70 (1)	
Unemployed	105 (1)	69 (2)	36 (1)	
COVID-19-related information				
Long COVID-19, sick leave ≥ 84 days, no, n (%)	8142 (91)	3416 (89)	4726 (93)	** < 0.001**
Yes, n (%)	793 (9)	417 (11)	376 (7)	
Length of sick leave in days during the 4 months post COVID-19				
Mean (± SD)	43.1 (25.6)	46.3 (27.4)	40.7 (24.0)	** < 0.001**^**#**^
Median (IQR)/min–max	35 (20)/1–122	36 (25)/1–122	33 (17)/2–122	

Variables with missing values n (%): country of birth, 6 (0.1%); education, 64 (0.7%); marital status, 7 (0.1%); children living at home, 7 (0.1%); ICD diagnosis codes in the year prior to COVID-19, 259 (2.9%); and employment status, 3 (0.03%)

**Abbreviations:** *COVID-19* coronavirus disease, *ICD* International Classification of Diseases, *IQR* interquartile range, *SD* standard deviation, *SEK* Swedish Krona

**Statistics:** Chi-square test and #Mann–Whitney U test. Bold text indicates statistically significant results at an alpha of 5%

### Association between pre-COVID-19 and COVID-19-related sick leave

**Fig. 2:**
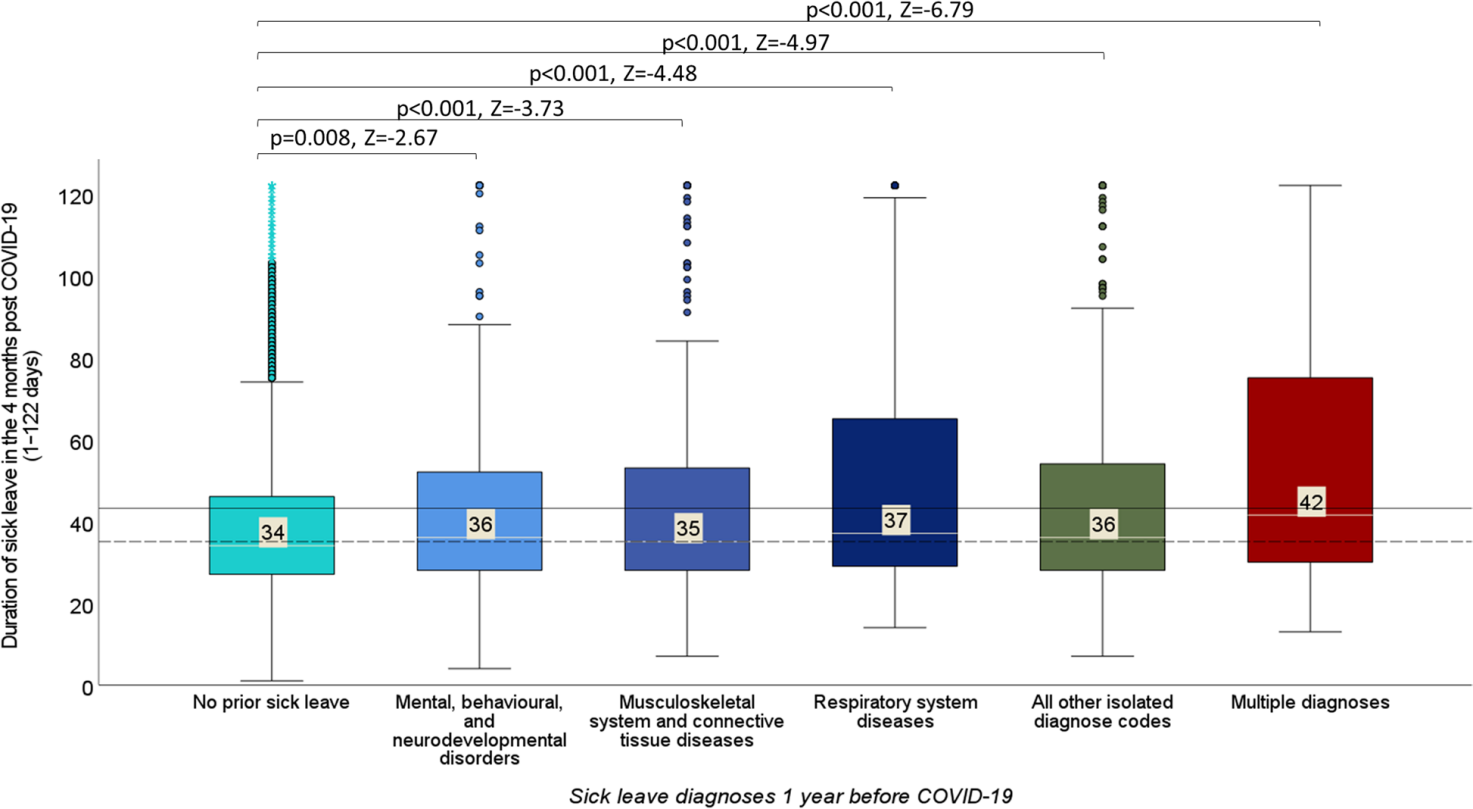
The duration of sick leave due to COVID-19 among individuals with different diagnoses 1 year before the COVID-19 diagnosis. Statistics: *p* and *Z* values correspond to the Mann–Whitney *U* test, the straight and dashed lines represent the mean and median lengths of sick leave in the study population, respectively

A difference was observed between the sick leave diagnoses in the year before the COVID-19 diagnosis and the median length of sick leave due to COVID-19 (Kruskal–Wallis test, *p* < 0.001). Significant differences were found between each diagnostic group when compared with the reference group with no prior history of sick leave (Fig. 2).

The multivariable model showed that, compared with the group without prior sick leave, the odds ratio (OR) was significantly elevated in the groups with musculoskeletal system and connective tissue diseases (OR: 1.08), respiratory system diseases (OR: 1.22), all other isolated diagnoses (OR: 1.08), and multiple diagnoses (OR: 1.30). Prior history of sick leave due to mental, behavioural, and neurodevelopmental disorders was not significantly associated with the length of sick leave due to COVID-19 during the first wave of the COVID-19 pandemic (Table 2).

**Table 2:** Multivariable model describing the factors associated with duration of sick leave in the 4 months during the first wave of the COVID-19 pandemic, *n* = 8602

Factors	β [SE]	OR [95% CI]	*p*-value
Intercept of the multivariable model	3.60 [0.03]	36.5 [34.3–38.9]	**0.000**
*Diagnostic groups in the year before the COVID-19, Ref.* No prior sick leave			
Mental, behavioural, and neurodevelopmental disorders	0.02 [0.03]	1.02 [0.96–1.08]	0.597
Musculoskeletal system and connective tissue diseases	0.08 [0.03]	1.08 [1.01–1.15]	**0.017**
Respiratory system diseases	0.20 [0.03]	1.22 [1.14–1.31]	** < 0.001**
All other isolated diagnoses	0.08 [0.03]	1.08 [1.03–1.14]	**0.003**
Multiple diagnoses	0.26 [0.04]	1.30 [1.20–1.41]	** < 0.001**
*Education. Ref. primary school (*≤ *9 years)*			
Secondary school (10–12 years)	− 0.05 [0.02]	0.95 [0.91–0.99]	**0.007**
Short university education (13–14 years)	0.02 [0.02]	1.02 [0.97–1.07]	0.425
Long university education (≥ 15 years)	0.03 [0.02]	1.03 [0.99–1.07]	0.143
*Civil status. Ref. married*			
Single	0.04 [0.01]	1.04 [1.01–1.06]	**0.004**
Divorced	0.03 [0.02]	1.03 [1.00–1.06]	0.053
Widow/widower	0.16 [0.05]	1.18 [1.07–1.30]	** < 0.001**
*Employment status. Ref. employed*			
Self-employed	0.11 [0.04]	1.12 [1.03–1.21]	**0.007**
Unemployed	− 0.06 [0.05]	0.95 [0.86–1.05]	0.295
Female sex	0.03 [0.01]	1.03 [1.01–1.06]	**0.011**
Age, per gained year (range 18–76 years)	0.002 0.000]	1.002 [1.001–.003]	** < 0.001**
Sick leave ≥ 28 days in the year before the COVID-19	0.04 [0.03]	1.04 [0.99–1.09]	0.161

**Statistics:** negative binomial regression, multivariable analysis. Bold text indicates statistically significant results at an alpha of 5%

**Abbreviations:** *COVID-19* coronavirus disease, *β* [SE], regression coefficients and standard errors, *OR* odds ratio, *CI* confidence interval

**Model metrics:** Akaike information criteria, 75,688; log-likelihood test: 37,826; omnibus test *p* = 0.000

### Subgroup analyses

**Fig. 3:**
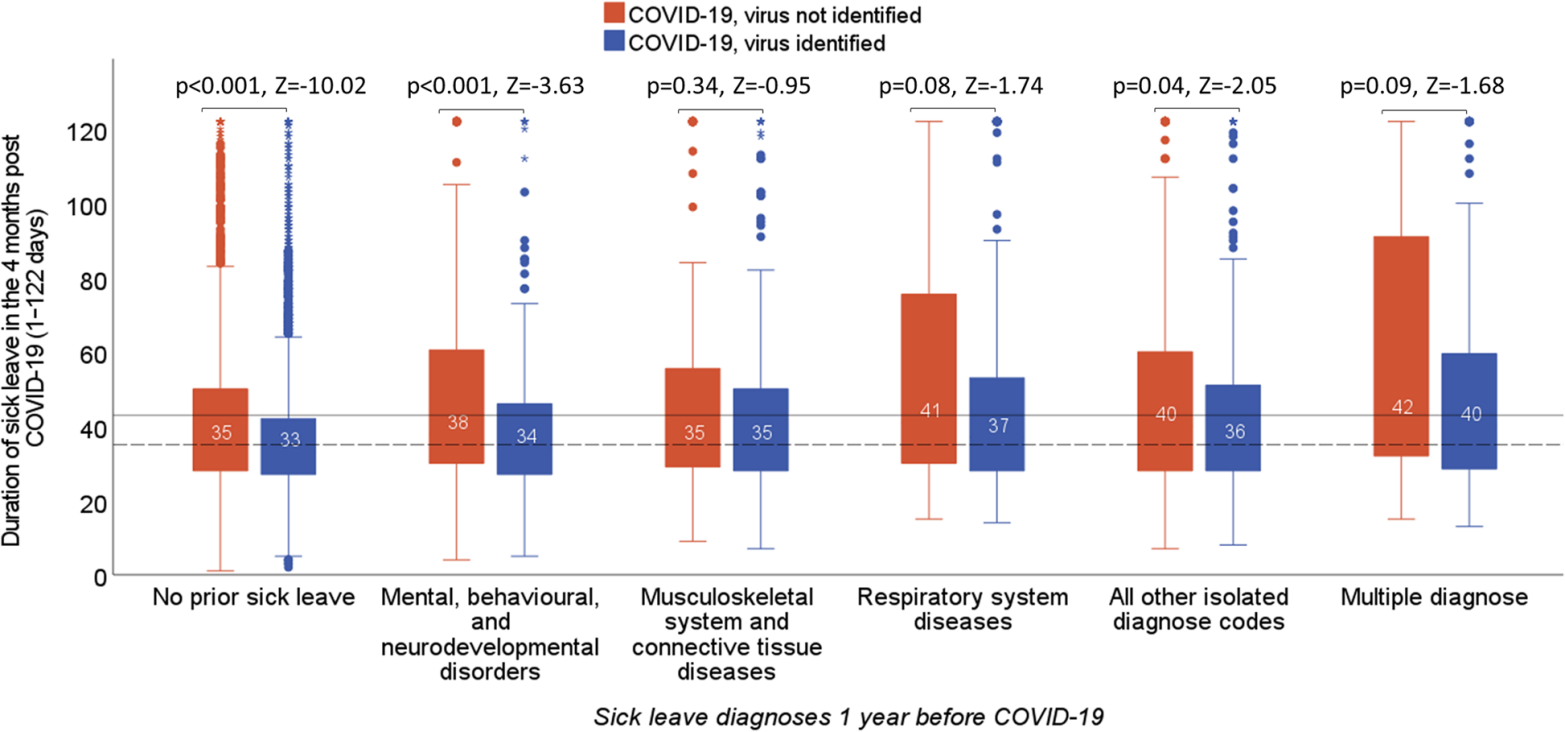
The duration of sick leave due to COVID-19 among individuals with and without confirmed SARS-CoV-2 infection. The results are stratified based on sick leave diagnoses from 1 year before the COVID-19 diagnosis. Statistics: *p* and *Z* values correspond to the Mann–Whitney *U* test, the straight and dashed lines represent the mean and median lengths of sick leave in the study population, respectively. COVID-19, coronavirus disease 2019; SARS-CoV-2, severe acute respiratory syndrome coronavirus 2

Subgroup analyses were performed on individuals with a COVID-19 diagnosis, with and without SARS-CoV-2 detection. The median length of sick leave was higher among individuals without SARS-CoV-2 detection compared with that for individuals with SARS-CoV-2 detection. However, this difference was significant only in individuals without prior sick leave; individuals with mental, behavioural, and neurodevelopmental disorders; and individuals with all other isolated diagnoses (Fig. 3).

### Subgroup with a COVID-19 diagnosis without SARS-CoV-2 detection

Compared with the group without prior sick leave, the sick leave duration was significantly increased in the group with respiratory diseases (OR: 1.21) and in the group with multiple diagnoses (OR: 1.29) (Table 3).

**Table 3:** Multivariable model describing the factors associated with duration of sick leave in the 4 months during the first wave of the COVID-19 pandemic, including subgroup of individuals with COVID-19 virus not identified, *n* = 3682

Factors	β [SE]	OR [95% CI]	*p*-value
Intercept of the multivariable model	3.72 [0.05]	41.1 [37.4–45.3]	**0.000**
*Diagnostic groups in the year before the COVID-19,* Ref. No prior sick leave			
Mental, behavioural, and neurodevelopmental disorders	0.03 [0.05]	1.03 [0.94–1.14]	0.507
Musculoskeletal system and connective tissue diseases	0.03 [0.05]	1.03 [0.94–1.14]	0.517
Respiratory system diseases	0.19 [0.05]	1.21 [1.09–1.35]	** < 0.001**
All other isolated diagnoses	0.04 [0.04]	0.04 [0.96–1.13]	0.355
Multiple diagnoses	0.26 [0.06]	1.29 [1.15–1.46]	** < 0.001**
*Education. Ref. primary school (*≤ *9 years)*			
Secondary school (10–12 years)	− 0.03 [0.03]	0.97 [0.92–1.03]	0.350
Short university education (13–14 years)	0.03 [0.03]	1.03 [0.96–1.10]	0.465
Long university education (≥ 15 years)	0.06 [0.03]	1.06 [1.00–1.13]	0.067
*Civil status. Ref. married*			
Single	0.03 [0.02]	1.03 [0.99–1.08]	0.099
Divorced	0.03 [0.02]	1.03 [0.99–1.08]	0.161
Widow/widower	0.15 [0.08]	1.17 [1.01–1.36]	0.046
*Employment status. Ref. employed*			
Self-employed	0.07 [0.05]	1.07 [0.97–1.19]	0.186
Unemployed	− 0.12 [0.07]	0.89 [0.78–1.01]	0.070
Female sex	0.06 [0.02]	1.06 [1.02–1.09]	**0.002**
Age, per gained year (range 18–76 years)	0.001 [0.000]	.001 [1.000–1.002]	0.534
Sick leave ≥ 28 days in the year before the COVID-19	0.05 [0.04]	1.06 [0.97–1.14]	0.190

**Statistics:** negative binomial regression, multivariable analysis. Bold text indicates statistically significant results at an alpha of 5%

**Abbreviations:** *COVID-19* coronavirus disease, *β* [SE], regression coefficient with standard error, *OR* odds ratio, *CI* confidence interval

**Model metrics:** The Akaike information criteria, 33,068; log-likelihood test: -16,516; omnibus test, p 0.001

### Subgroup with a COVID-19 diagnosis with SARS-CoV-2 detection

Compared with that in the group without prior sick leave, the sick leave duration was significantly increased in the groups with musculoskeletal system and connective tissue diseases (OR: 1.12), respiratory system diseases (OR: 1.24), other isolated diagnoses (OR: 1.13), and multiple diagnoses (OR: 1.29) (Table 4).

**Table 4:** Multivariable model describing the factors associated with duration of sick leave in the 4 months during the first wave of the COVID-19 pandemic, including subgroup of individuals with confirmed SARS-CoV-2 infection, *n* = 4920

Factors	β [SE]	OR [95% CI]	*p*-value
Intercept of the multivariable model	3.50 [0.04]	33.1[30.5–36.0]	**0.000**
*Diagnostic groups in the year before the COVID-19. Ref. No prior sick leave*			
Mental, behavioural, and neurodevelopmental disorders	− 0.00 [0.04]	0.99 [0.92–1.08]	0.978
Diseases of the musculoskeletal system and connective tissue	0.12 [0.04]	1.12 [1.04–1.21]	**0.003**
Diseases of the respiratory system	0.21 [0.04]	1.24 [1.14–1.34]	** < 0.001**
All other isolated diagnoses	0.12 [0.03]	1.13 [1.06–1.20]	** < 0.001**
Multiple diagnoses	0.26 [0.05]	1.29 [1.17–1.44]	** < 0.001**
*Education. Ref. primary school (*≤ *9 years)*			
Secondary school (10–12 years)	− 0.06 [0.03]	0.94 [0.89–0.99]	**0.016**
Short university education (13–14 years)	0.003 [0.03]	1.003 [0.95–1.06]	0.921
Long university education (≥ 15 years)	0.001 [0.03]	1.001 [0.95–1.06]	0.963
*Civil status. Ref. married*			
Single	0.04 [0.02]	1.04 [1.00–1.07]	**0.035**
Divorced	0.02 [0.02]	1.01 [0.98–1.06]	0.452
Widow/widower	0.18 [0.06]	1.19 [1.06–1.34]	**0.003**
*Employment status. Ref. employed*			
Self-employed	0.13 [0.06]	1.14 [1.01–1.29]	**0.040**
Unemployed	− 0.004 [0.09]	0.99 [0.84–1.19]	0.965
* Female sex*	0.04 [0.02]	1.04 [1.01–1.07]	**0.021**
Age, per gained year (range 18–76 years)	0.003 [0.000]	1.003 [1.002–1.005]	** < 0.001**
Sick leave ≥ 28 days in the year before the COVID-19	0.01 [0.03]	1.01 [0.95–1.08]	0.654

**Statistics:** negative binomial regression, multivariable analysis. Bold text indicates statistically significant results at an alpha of 5%

**Abbreviations:** *COVID-19* coronavirus disease, *β* [SE], regression coefficient with standard error, *OR* odds ratio, *CI* confidence interval

**Model metrics:** The Akaike information criteria, 42,484; log-likelihood test: -21,1145; omnibus test, *p* 0.000

## Discussion

This study contributes to a better understanding of the underlying causes of prolonged sick leave in individuals with COVID-19. The results of this nationwide registry-based study of non-hospitalised individuals with COVID-19 revealed a median length of COVID-19-related sick leave of 35 days. The sick leave duration was longer in individuals with prior sick leave diagnoses of musculoskeletal system and connective tissue diseases, respiratory system diseases, all other isolated diagnoses, and multiple diagnoses. Sociodemographic characteristics of the participants were also significant predictors.

The sick leave could be an indicator of undesirable health outcome, including extended work absence [16, 17]. Many individuals have both short- and long-term residual difficulties after COVID-19 [18, 19]. In the present study, during the year before COVID-19 diagnosis, nearly one-fourth of the study population had sick leave-related diagnoses and 17% had a sick leave duration ≥ 28 days. Furthermore, we found that individuals with a history of sick leave in the year prior to the COVID-19 diagnosis had higher odds of prolonged sick leave due to COVID-19, particularly in the groups with prior respiratory system diseases and multiple diagnoses. In this study, the respiratory system disease group comprised individuals with various ICD codes, such as influenza, inflammation of the respiratory system, pneumonia, and chronic obstructive pulmonary disease. People with pre-existing respiratory diseases have a high risk of severe COVID-19 [20]. Moreover, SARS-CoV-2 infection is correlated with specific cells of the respiratory system [21]. The combination of premorbid fragility of the respiratory tract and the pathogenic mechanism of SARS-CoV-2 could lead to more severe health problems, which might have been reflected as prolonged sick leave in this study. Our findings are in line with those of a previous Finnish study that found that precedent sick leave was a predictor of future sick leave episodes with a stronger association for longer sick leave episodes [22]. Another study among Brazilian hospital employees found that previous sick leave increased the risk of subsequent sick leave episodes which differed by diagnosis, although all the diagnoses had a significant effect [23]. Furthermore, the group with multiple diagnoses comprised individuals who had been on sick leave due to various diagnoses in the year prior to COVID-19 diagnosis. The number and types of underlying conditions have been associated with COVID-19 severity [11, 24–26], as we observed in this study. Individuals with multiple diagnoses may have lower overall resilience and thus, the COVID-19 recovery, as expressed by the duration of sick leave in this study, was prolonged. Surprisingly, having a prior diagnosis of mental, behavioural, and neurodevelopmental disorders was not significantly associated with the length of sick leave related to COVID-19. Among the general population, sick leave due to mental disorders is common [27]. Moreover, individuals with a history of sick leave due to mental disorders had a high risk for recurrent sick leave, disability pension, and unemployment [28]. However, variations among the diagnoses were found [28]. This difference could be because several diagnostic codes were merged under the group of mental, behavioural, and neurodevelopmental disorders, and statistical significance could not be obtained due to variability in the duration of sick leave associated with different diagnoses.

The length of sick leave differed between individuals with and without SARS-CoV-2 detection. The median sick leave duration was 3 days longer for participants without compared with participants with SARS-CoV-2 detection. In addition, in the subgroup of individuals in whom SARS-CoV-2 was not detected, participants with respiratory system diseases and multiple diagnoses necessitating sick leave prior to COVID-19 had higher odds of longer sick leave durations than those of participants without these diagnoses. In the subgroup in which SARS-CoV-2 was detected, all groups except those with individuals with mental, behavioural, and neurodevelopmental disorders had higher risk of longer sick leave than that of individuals without sick leave in the year prior to the COVID-19 diagnosis. At the beginning of the pandemic, emergency ICD codes were activated for COVID-19 and the diagnoses were made based on confirmed positive laboratory tests (U07.1) or clinical/epidemiological symptoms (U07.2). The difference in sick leave durations might be because the group without SARS-CoV-2 detection comprised participants with less reliable diagnoses. In addition, participants without SARS-CoV-2 detection differed from participants with SARS-CoV-2 detection [12]. In the early stages of the pandemic, the proportion of the population that was tested for SARS-CoV-2 infection varied throughout Sweden [1] and the guidelines for sick leave were somewhat vague. Although workers were encouraged to stay at home if they developed any related symptoms, the time point for a safe return to work was undefined [29]. Together, these factors could explain the difference in sick leave durations between individuals with and without SARS-CoV-2 detection.

Females had slightly higher odds of COVID-19-related sick leave than males among all study participants and in the subgroup analyses. One explanation may be the overall tendency of more women than men to be on sick leave in Sweden [7], specifically if not receiving inpatient care due to COVID-19. In general, individuals who required inpatient care due to COVID-19, received more sickness benefits [8]. In this study, individuals who were hospitalised for COVID-19 were excluded, as they were expected to have more severe COVID-19 and prolonged sick leave. Dropout analyses revealed that those excluded were predominantly males. Sex differences related to COVID-19 outcomes have been reported, with more severe outcomes in men [20, 30, 31]. Therefore, it is possible that our results were positively skewed towards females.

Sociodemographic factors such as education, employment status, and marital status were related to the length of sick leave during the 4 months of follow-up after COVID-19 diagnosis. However, the results of the regression analyses varied by SARS-CoV-2 detection among all study participants and in subgroup analyses stratified by SARS-CoV-2 detection. In this study, individuals with a secondary school education tended to have shorter sick leave durations than that of participants in the reference group. Frontline workers (individuals who cannot feasibly work from home and must provide their labour in person) often [32] report higher job insecurity and low income [33, 34]. Moreover, some workers might have been employed hourly. Together, these factors may affect the length of COVID-19-related sick leave. Compared with individuals who were living in partnership, individuals who were single, widowed, or divorced had higher odds of longer sick leave durations due to COVID-19. Thus, we speculated that because these individuals had a limited possibility of load alleviation with household tasks, and possibly due to COVID-19-related fatigue, that recovery time was prolonged.

This study has several strengths and limitations. The major strength of this study is the large nationwide data from all non-hospitalised individuals with sickness benefits due to COVID-19. Few individuals were excluded; therefore, it can be assumed that the study population was representative of the group of non-hospitalised individuals who had COVID-19 in the first pandemic wave. In addition, the sick leave period due to COVID-19 was considered the number of days with sickness benefits and had to include at least one registered COVID-19 diagnosis; other included diagnoses were fever, unspecified viral infection, or other infections with similar symptoms to COVID-19. However, some participants might have been mis-classified as having COVID-19. However, we wanted include all possible individuals with sick leave due to COVID-19, and at the beginning of the pandemic, testing for COVID-19 was not readily available and the diagnostic criteria were somewhat vague.

The inclusion of individuals without SARS-CoV-2 detection in the analysis might have led to unreliable data and results due to the nature of the diagnoses, as the diagnosis may have been less reliable. The major limitation of this study is the lack of information regarding COVID-19 severity, COVID-19-related symptoms, and symptom duration. Moreover, the pre-COVID-19 sick leave diagnoses were combined into large diagnostic groups; therefore, we could not analyse the previous diagnoses in more detail. Finally, information about the type of work, work demands, and work tasks was unavailable. These factors may all contribute to the length of sick leave [35].

## Conclusion

The results of this nationwide registry-based study indicate that individuals with sick leave in the year prior to the COVID-19 diagnosis are more prone to longer sick leave durations due to COVID-19. This knowledge is important for decision making by various authorities and healthcare professionals regarding sick leave and sickness benefits. In addition, identifying groups of individuals with high odds of long-term sick leave due to COVID-19 can facilitate the development of targeted support and rehabilitation [19].

## Supplementary Information

**Additional file 1: Table S1.** Overview of the study variables, their categories, and primary roles in the analyses. **Table S2.** Correlation coefficients between the study variables. **Fig. S1.** Directed acyclic graph showing factors that might confound the relationship between sick leave diagnosis groups 1 year prior to the COVID-19 diagnosis and the length of sick leave in the 4 months after a COVID-19 diagnosis.

## Data Availability

According to Swedish regulations, the dataset generated within this study’s framework cannot be made publicly available for ethical and legal reasons. The research data can be made available on reasonable request to the corresponding author.

## Abbreviations

AIC: Akaike information criterion
CI: Confidence interval
COVID-19: Coronavirus disease 2019
DAG: Directed acyclic graph
ICD: International Classification of Diseases
IQR: Interquartile range
OR: Odds ratio
SD: Standard deviation
SSIA: Swedish Social Insurance Agency
