# Supplementary material for: Impact of pre-pandemic sick leave diagnoses on the length of COVID-19-related sick leave: a nationwide registry-based study
Source: BMC Public Health. 2023 Jan 29;23:195. doi: 10.1186/s12889-023-15115-x (PMC9884157; doi:10.1186/s12889-023-15115-x)
Supplement: Supplementary file 1 — Additional file 1: Table S1. Overview of the study variables, their categories, and primary roles in the analyses. Table S2. Correlation coefficients between the study variables. Fig. S1. Directed acyclic graph showing factors that might confound the relationship between sick leave diagnosis groups 1 year prior to the COVID-19 diagnosis and the length of sick leave in the 4 months after a COVID-19 diagnosis. [file 12889_2023_15115_MOESM1_ESM.docx]

Supplementary Information

**Impact of pre-pandemic sick leave diagnoses on the length of COVID-19-related sick leave: A nationwide registry-based study**

Tamar Abzhandadze, OT, PhD^1,2^*, Emma Westerlind, MD, PhD^1^, Hanna C. Persson, PT, PhD^1,2^

^1^Institute of Neuroscience and Physiology, The Sahlgrenska Academy, University of Gothenburg, Gothenburg, Sweden

^2^Department of Occupational Therapy and Physiotherapy, Sahlgrenska University Hospital, Gothenburg, Sweden

*Corresponding author: Tamar Abzhandadze, E-mail: tamar.abzhandadze@gu.se

Table S1. Overview of the study variables, their categories, and primary roles in the analyses

| **Variables** | **Categories and coding** | **Level and role** | **Inclusion in the regression model** |
| --- | --- | --- | --- |
| COVID-19 sick leave, total (days) | Continuous | **Outcome (count data)** |  |
| ICD diagnosis groups 1 year before commencement of COVID-19 sick leave | 0: No prior sick leave | **Primary explanatory variable (nominal)** |  |
|  | 1: Mental, behavioural, and neurodevelopmental disorders |  |  |
|  | 2: Musculoskeletal system and connective tissue diseases |  |  |
|  | 3: Respiratory system diseases |  |  |
|  | 4: All other isolated diagnoses |  |  |
|  | 5: Multiple diagnoses |  |  |
| Sex | 1: Male | Potential explanatory (binary) | **Ѵ** |
|  | 2: Female |  |  |
| Age, years | Range 18–74 | Potential explanatory (continuous) | **Ѵ** |
| Country of birth | 1: Sweden | Potential explanatory (nominal) |  |
|  | 2: Nordic countries, except for Sweden |  |  |
|  | 3: European countries, except for the Nordic countries |  |  |
|  | 4: Countries of Asia |  |  |
|  | 5: All other countries |  |  |
| Sick leave 1 year prior to COVID-19 diagnosis | 1: No | Potential explanatory (binary) | **Ѵ** |
|  | 2: Yes, ≥28 days |  |  |
| Education level | 1: Primary school (≤9 years) | Potential explanatory (ordinal) | **Ѵ** |
|  | 2: Secondary school (10–12 years) |  |  |
|  | 3: Short university education (13–14 years) |  |  |
|  | 4: Long university education (≥15 years) |  |  |
| Civil status | 1: Married | Potential explanatory (nominal) | **Ѵ** |
|  | 2: Single |  |  |
|  | 3: Divorced |  |  |
|  | 4: Widow/widower |  |  |
| Children living at home | 0: No | Descriptive (binary) |  |
|  | 1: Yes |  |  |
| Children ≤18 years old living at home | 0: No | Potential explanatory (binary) |  |
|  | 1: Yes |  |  |
| Type of employment | 1: Employed | Potential explanatory (nominal) | **Ѵ** |
|  | 2: Self-employed |  |  |
|  | 3: Unemployed |  |  |
| Income in 10,000 SEK | Continuous | Descriptive (scale) |  |
| COVID-19 diagnosis | 1: SARS-CoV-2 not detected, U07.2 | Descriptive and used for subgroup analyses |  |
|  | 2: SARS-CoV-2 detected, U07.1 |  |  |
| Long COVID-19 | 1: No long COVID-19, sick leave ≤83 days | Descriptive |  |
|  | 2: Long COVID-19, sick leave ≥84 days |  |  |

COVID-19, coronavirus disease; SARS-CoV-2, severe acute respiratory syndrome coronavirus 2; SEK, Swedish Krona; ICD, International Classification of Diseases.

Table S2. Correlation coefficients between the study variables

|  | | | Length of sick leave post COVID-19 (range 1–122 days) | | ICD diagnosis codes 1 year prior to  COVID-19 | | Sick leave 1 year prior to COVID-19,  ≥28 days | | Sex | | Age (18–76 years) | | Country of birth | | Education | | Civil status | | Children ≤18 years old living at home |
| --- | --- | --- | --- | --- | --- | --- | --- | --- | --- | --- | --- | --- | --- | --- | --- | --- | --- | --- | --- |
|  | ICD diagnosis codes 1 year prior to COVID-19 (range 1–122 days) | .097^**^ | |  | |  | |  | |  | |  | |  | |  | |  | |
|  |  |  | |  | |  | |  | |  | |  | |  | |  | |  | |
|  | Sick leave 1 year prior to COVID-19, ≥28 days | .077^**^ | | **.798^**^** | |  | |  | |  | |  | |  | |  | |  | |
|  |  |  | |  | |  | |  | |  | |  | |  | |  | |  | |
|  | Sex | .038^**^ | | .083^**^ | | .067^**^ | |  | |  | |  | |  | |  | |  | |
|  |  |  | |  | |  | |  | |  | |  | |  | |  | |  | |
|  | Age, range (18–76 years) | .054^**^ | | .060^**^ | | .039^**^ | | .041^**^ | |  | |  | |  | |  | |  | |
|  |  |  | |  | |  | |  | |  | |  | |  | |  | |  | |
|  | Country of birth | −.001 | | −.013 | | −.028^**^ | | −.049^**^ | | −.062^**^ | |  | |  | |  | |  | |
|  |  |  | |  | |  | |  | |  | |  | |  | |  | |  | |
|  | Education | .022^*^ | | −.043^**^ | | −.035^**^ | | .150^**^ | | −.018 | | −.098^**^ | |  | |  | |  | |
|  |  |  | |  | |  | |  | |  | |  | |  | |  | |  | |
|  | Civil status | .027^**^ | | .026^*^ | | .028^**^ | | .018 | | −.067^**^ | | −.047^**^ | | −.052^**^ | |  | |  | |
|  |  |  | |  | |  | |  | |  | |  | |  | |  | |  | |
|  | Children ≤18 years old living at home | −.020 | | −.003 | | .000 | | .062^**^ | | −.309^**^ | | .110^**^ | | .089^**^ | | −.186^**^ | |  | |
|  |  |  | |  | |  | |  | |  | |  | |  | |  | |  | |
|  | Employment status | −.002 | | .028^**^ | | .014 | | −.065^**^ | | .043^**^ | | .038^**^ | | −.043^**^ | | .006 | | −.005 | |
|  |  |  | |  | |  | |  | |  | |  | |  | |  | |  | |
|  | Statistics: r_s_, Spearman’s rank order correlation coefficients; Correlation is significant at ** 0.01 level and * 0.05 level. Abbreviations: ICD, International Classification of Diseases. | | | | | | | | | | | | | | | | | | |


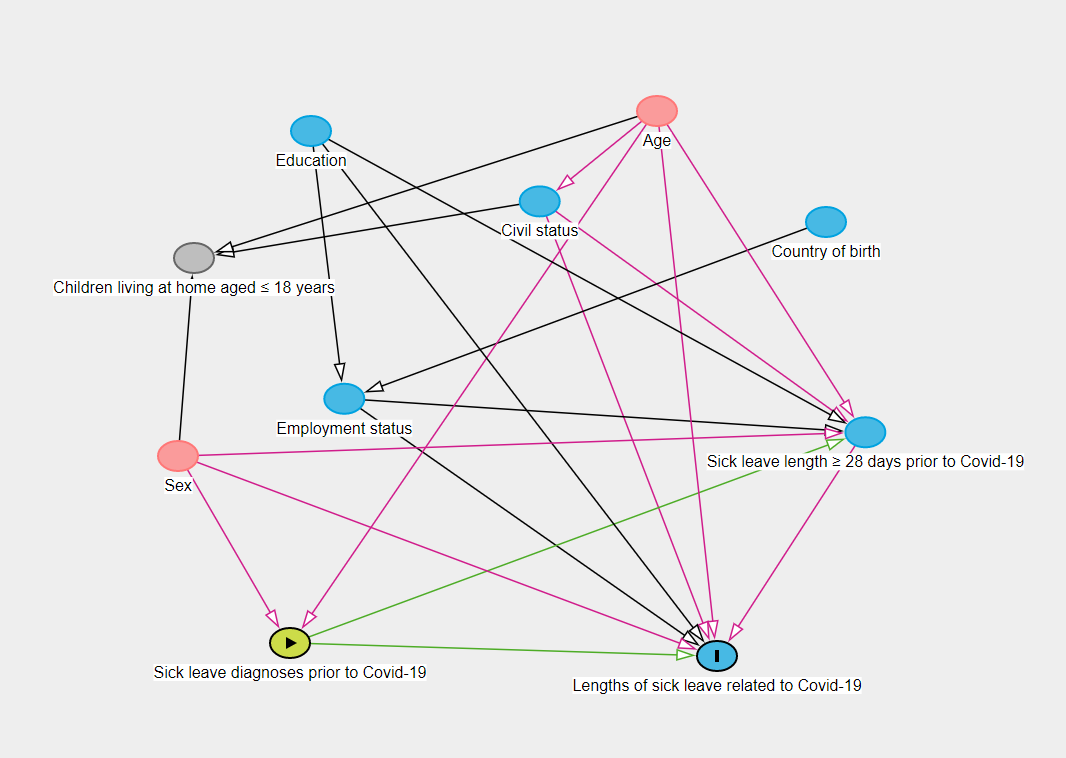


**Fig. S1** Directed acyclic graph showing factors that might confound the relationship between sick leave diagnosis groups 1 year prior to the COVID-19 diagnosis and the length of sick leave in the 4 months after a COVID-19 diagnosis.
